# *Helicobacter pylori* resistance to metronidazole and its association with virulence factors in a Moroccan population

**DOI:** 10.11604/pamj.2022.42.144.33217

**Published:** 2022-06-22

**Authors:** Essaidi Imane, Bounder Ghizlane, Jouimyi Mohamed Reda, Boura Hasna, Elyounsi Ilhame, Benomar Hakima, Badre Wafa, Zerouali Khalid, Maachi Fatima

**Affiliations:** 1 Laboratory of Helicobacter Pylori and Gastric Pathologies, Pasteur Institute of Morocco, Casablanca 20360, Morocco,; 2 Faculty of Medicine of Casablanca, Ibn Rochd University Hospital Center, Hassan II University, Casablanca, Morocco,; 3 Faculty of Medicine, University of Tours, Tours, France,; 4 Laboratory of Histo-Cytopathology, Pasteur Institute of Morocco, Casablanca 20360, Morocco,; 5 Gastroenterology Department, Ibn Rochd University Hospital Center, Casablanca 20360, Morocco

**Keywords:** *Helicobacter pylori*, resistance, metronidazole, VacA, CagA, eradication

## Abstract

**Introduction:**

surveillance data on Helicobacter pylori (H. pylori) antibiotic susceptibilities in Morocco are limited, despite resistance being the key factor in treatment failure. Virulence factors of H. pylori are associated with carcinogenesis and may be also associated with the efficacy of treatment. The aim of our study is to determine the prevalence of H. pylori metronidazole resistance in a Moroccan population infected with H. pylori and to study the impact of their virulence factors CagA and VacA on their resistance to metronidazole.

**Methods:**

the susceptibility to metronidazole of 185 isolates was determined by PCR. The isolates were also genotyped for CagA and VacA genes by PCR.

**Results:**

the metronidazole resistance rate was 62.70%. No association between resistance to metronidazole and social factors was detected. Regarding the virulence factors, we remarked that the moderate virulent strains s1/m2/i1/d1 with a CagA negative were the most resistant to metronidazole with a rate of 84% compared to the less virulent strains bearing the CagA negative VacA s2m2i2d2 genotype with a rate of 58% and the high virulent strains s1/m1/i1/d1-CagA positive with a rate of 47.06%.

**Conclusion:**

our study revealed a very high prevalence of resistance to metronidazole in our population. The resistance ability of H. pylori maybe affected by its virulence intensity. H. pylori eradication regimens should therefore be reevaluated in this setting.

## Introduction

*Helicobacter pylori (H. pylori)* is a Gram-negative, microaerophilic, spiral bacterium that colonizes the stomach of approximately half the world´s population [1]. In Morocco, the prevalence of *H. pylori* infection exceeds 70% [2,3]. This bacterium is an important risk factor in chronic gastritis, peptic ulcer disease, gastric carcinoma, and mucosa-associated lymphoid tissue (MALT), and in 1994, was classified as a group 1 carcinogen. Different regimens to eradicate this microorganism has been used in clinical practice. Metronidazole has frequently been used as a component in these treatment regimens. Metronidazole is also used in the treatment of other diseases, such as gynecological infections, and has contributed to the increased antibiotic resistance of *H. pylori* [4,5].

Since 2016, in Morocco and according to the fifth edition of the Maastricht Consensus Conference, the first-line therapy for treating *H. pylori* infection consists of a bismuth-based quadruple therapy namely PYLERA (a three-in-one capsule containing bismuth salt, tetracycline, and metronidazole) proton pump inhibitor (PPI), or Concomitant quadruple therapy, containing a PPI, amoxicillin, clarithromycin, and metronidazole given twice a day for 10-15 days. Metronidazole resistance has been attributed to key mutations in a relatively small number of nucleotide and amino acid sequences. Amino acid substitutions in the sequences of oxygen-independent NADPH nitroreductase (RdxA) and NADH flavin oxidoreductase (FrxA) have been reported to be associated with metronidazole resistance [6].

The most commonly studied virulence factors in *H. pylori* are encoded by the cytotoxin-associated gene A (CagA) and the vacuolating associated gene A (VacA). The CagA, which is the marker for the presence of the pathogenicity island (Cag PAI), is the most studied putative virulence factor. Based on the presence or the absence of the CagA gene, *H. pylori* strains can be divided into CagAA-positive or CagA-negative. The CagA protein acts as an oncoprotein by perturbing multiple host signaling pathways (cellular proliferation, cell elongation, and scattering) that predispose the acquisition of neoplastic traits by gastric epithelial cells [7-9]. Concerning the VacA, there are at least 8 variable regions. In the signal (s) region, in which one of two alleles can be present: s1 or s2, and in the middle (m) region, in which one of two alleles can be present; m1 or m2 [10]. Recently Two new regions were described: the i1 and i2 subtypes ("i" for Intermediate) and d1 or d2 subtypes ("d" region for deletion) [11]. The variable structure, resulting in different allelic arrangements in the gene, is related to differences in cytotoxin production and distinct clinical outcomes of *H. pylori* infection [12]. For example, a recent study performed at our institute has demonstrated a correlation between the presence of genetic polymorphisms in the VacA gene and the severity of gastritis [13]. The aim of this study was to analyze metronidazole resistance and virulence-factor genotypes in our strains and to evaluate if there was any relationship between metronidazole resistance and virulence-factor genotypes.

## Methods

**Study design**: a cross-sectional study was conducted to determine the prevalence of *H. pylori* resistance to metronidazole and its association with virulence factors in a Moroccan population.

**Study population and data collection**: the present study was carried out on 195 patients with various gastritis from departments of Gastroenterology consulting at Ibn Rochd University Hospital Center, Casablanca (Gastroenterology and Oncology departments). The patients were 89 males and 106 females, ranging from 18 to 86 years old (mean age: 49.86 ± 16.39 years). All patients benefited from an upper gastrointestinal endoscopy. Three biopsies (1 antrum, 1 fundus, and 1 lesser curvature) were sampled from all patients from 2017 to 2020 and used for histological examination and molecular detection. The biopsies were fixed in 10% formalin and taken to the Histo-Cytopathology Laboratory in Pasteur Institute of Morocco, where they were embedded in paraffin blocks, sectioned, and stained in hematoxylin and eosin for conventional histopathology examination of gastric mucosal lesions and *H. pylori* infection. Classification of gastric lesions was done according to the Sydney system. Of all patients, 116 had active chronic gastritis, 51 had atrophic gastritis and 28 had intestinal metaplasia. Clinical information about demographic characteristics of participants including age, sex, living area, smoking, and alcohol habits) was collected using a structured survey. All participants were informed about their inclusion in the study and agreed to it in writing form. The study protocol has been performed in accordance with the ethical standards of Helsinki and was approved by the committee of the Pasteur Institute of Morocco.

**DNA extraction**: whole genomic DNA was extracted from gastric biopsies using a genomic DNA extraction kit (Isolate Genomic DNA Kit, Bioline) according to the manufacturer´s instructions. Then it was stored at -20°C until use.

***H. pylori* detection**: the ure C gene (296 bp) was amplified to detect *H. pylori* infection, using specific primers (Table 1) [14-19]. PCR reaction mixture was prepared with 0.5 mM dNTPs, 1.5 mM MgC2, 0.5 μM of each primer, one U of DNA Polymerase (MyTaq DNA Polymerase, BioLine), and 300 ng of DNA in a final volume of 20 µL. PCR thermocycling conditions were: 1 cycle at 95°C for 1 min, 35 cycles at 95°C for 15 s, 55°C for 30 s, 72°C for 30 s, and a final extension cycle at 72°C for 7 min.

**Table 1:** primers used in this study

Primer name	Primer sequence 5-3	Band size (bp)	Reference
ureC-F	AAGCTTTTAGGGGTGTTAGGGGTTT	296	[14]
ureC-R	AAGCTTACTTTCTAACACTAACGC		
rdxA1	Forward 5-AATTTGAGCATGGGGCAGA-3	850	[15]
rdxA2	Reverse 5-GAAACCGCTTGAAAACACCCCT-3		
CagA-F	GATAACAGGCAAGCTTTGAGG	349	[16]
CagA-R	GTGCAAAAGATTGTTTGGCAGA		
s(s1/s2) VAI-F	ATGGAAATACAACAAACACAC	s1 = 259	[17]
s(s1/s2) VAI-R	CTGCTTGAATGCGCCAAAC	s2 = 286	
m(m1/m2) VAG-F	CAATCTGTCCAATCAAGCGAG	m1 = 567	[17]
m(m1/m2) VAG-R	GCGTCAAAATAATTCCAAGG	m2 = 642	
i1 VACF1	GTTGGGATTGGGGGAATGCCG	426	
i1 C1R	TTAATTTAACGCTGTTTGAAG		[18]
i2 VACF1	GTTGGGATTGGGGGAATGCCG	432	
i2 C2R	GATCAACGCTCTGATTTGA		
d(d1/d2)VAS-5F	ACTAATATTGGCACACTGGATTTG	d1 = 367-379	[19]
d(d1/d2)VAGF-R	CTCGCTTGATTGGACAGATTG	d2 = 298	

**Metronidazole susceptibility detection**: deletion of a 200-bp fragment from the rdxA gene is one of the molecular mechanisms of resistance to metronidazole. To determine the gene deletion, the PCR method using specific primers was performed (Table 1) [14-19]. In the case of non-mutated strains, the amplification of this gene by PCR leads to the production of an 850-bp fragment. In the case of the defective gene, the PCR leads to the production of a 650-bp fragment. The rdxA gene was amplified by PCR in a total volume of 20 µL of reaction mix included: 5 µl of genomic DNA, 0.8 µM of each primer, 12,2 µl of H2O, and 0.2 µl of Taq polymerase (BIOLINE). Thermocycling conditions of PCR were as follows: initial denaturation at 95°C for 1 minute, followed by 35 cycles of 95°C for 15 seconds, 58°C for 30 sec, 72°C for 30 seconds, and a final extension at 72°C for 7 minutes. The PCR products were analyzed by 1.5% agarose gel electrophoresis.

### Virulence factors detection

**Detection of CagA gene**: according to the presence or absence of the CagA gene, *H. pylori* are classified into virulent CagA positive strains and non-virulent CagA negative strains. The CagA gene had been detected by PCR using specific primers (Table 1) [14-19]. PCR reaction mixture was prepared with 0.5 mM dNTPs, 1.5 mM MgCl 2, 0.2 µM of each dNTP, 1 U of DNA polymerase (MyTaq DNA Polymerase, BioLine), and 300 ng of genomic DNA in a final volume of 20 μL. PCR cycling conditions were: 1 cycle at 95°C for 1 min, 35 cycles at 95°C for 15 s, 57°C for 1 min, 72°C for 1 min, and a final extension at 72°C for 7 min.

**Genotyping of VacA regions**: the VacA regions (s, m, I, d) of strains were genotyped by PCR using specific primers (Table 1) [14-19]. For VacA s and m regions, the PCR reactions mixtures were prepared with 0.5 mM dNTPs, 1.5 mM MgCl2, 0.5 µM of each primer (Table 1) [14-19], 1 U of MyTaq DNA Polymerase (MyTaq DNA Polymerase, BioLine) and 300 ng of DNA in a final volume of 20 μL. For VacA i and d regions, the PCR reactions mixtures were prepared with 0.75 mM dNTPs, 2.25 mM MgCl2, 0.4 µM of each primer (Table 1) [14-19], 1 U of MyTaq DNA Polymerase (MyTaq DNA Polymerase, BioLine) and 300 ng of DNA in a final volume of 20 µL.

**Statistical analysis**: data entry and analysis were performed using RStudio software. The descriptive data are presented as frequencies and means ± standard deviations (SD). The differences between the groups were analyzed with t-test or Wilcox test for continuous variables, and Chi-square test or Fisher´s exact test for categorical variables. The differences were considered significant at p < 0.05.

## Results

**Description of the population studied**: a total of 195 gastric biopsies were obtained from patients with a histopathological diagnosis of chronic gastritis. Of all patients, 59.5% (116/195) had active chronic gastritis, 26.2% (51/195) had atrophic gastritis and (14.4%) (28/195) had intestinal metaplasia. The mean age of patients was 49.86 ± 16.39 (range of 20-86years). The rate of female patients was 54.4% (106/195) and the rate of male patients was 45.6% (89/195). Of 195 patients, *H. pylori* infection was detected in 185 individuals (94.9%) based on the detection of ure C gene by PCR technique.

**The overall prevalence of metronidazole resistance and social factors**: the study showed that 62.7% (116/185) of patients are infected with *H. pylori* strains resistant to Metronidazole while 37.3% (69/185) are infected with Metronidazole-susceptible strains. The distribution of metronidazole resistance according to gender showed that there is no significant difference between female and male patients (63.3% resistant strains for women and 62.7% for men, p-value = 0.98). Regarding the factor of age, we found that the age group of [61-90] was the most infected by metronidazole-resistant strains with a rate of 74.4% followed by the age group of [51-60] (71.1%). However, the difference was not statistically significant between the groups (p-value = 0.16). Regarding the living area, the metronidazole resistance was a little more detected in the rural area (64%) compared to the urban area (62%), but the difference was not statistically significant (p-value = 0.86). The study of the impact of tobacco and the consumption of alcohol on the risk of metronidazole resistance showed that there was no association between the *H. pylori* infection and the consumption of tobacco or alcohol (p-value = 1, p-value = 0.93 respectively) (Table 2).

**Table 2:** prevalence of metronidazole resistance and epidemiological factors

	Metronidazole-resistance (%)	Metronidazole-susceptibility (%)	P-value
**Factors(n)**			
**Gender**			
Men(87)	54(62.70)	33(37.93)	0.98
Women(98)	62(63.26)	36 (36.74)	
**Age**			
18-40(54)	35(64)	19(36)	
41-50 (71)	19(43.18)	25(56.82)	0.16
51-60(42)	30(71.14)	12(28.86)	
61-90(43)	32(74.41)	11(25.59)	
**The living area**			
Rural(59)	38(64)	21(36)	0.86
Urban (126)	78(60.1)	48(39.9)	
**Tobacco**			
yes(26)	17(65)	9(34.62)	
No(159)	99(62.23)	60(37.77)	0.9312
**Alcohol**			
yes(16)	10(62.25)	6(37.75)	
No(169)	106(62.72)	63(37.28)	1
**Total**(185)			

### The association of metronidazole resistance with virulence factors of *H. pylori*

**Relationship between *H. pylori* strains and gastric lesions**: previous work was carried out in our laboratory on the impact of virulence factors of *H. pylori* on gastric carcinogenesis which has shown that depending on the CagA-VacA genotypes, the strains were divided into high virulent strains VacA (s1/m1/i1/d1) CagA positive, responsible for the increased risk of developing intestinal metaplasia, moderate virulent strains VacA (s1/m2/i1/d1) CagA negative, most common in atrophic gastritis, and the less virulent strains VacA (s2/m2/i2/d2) CagA negative, most detected in chronic gastritis [20].

**Relationship between *H. pylori* strains and metronidazole resistance**: to evaluate the relationship between drug susceptibility and virulence-factor genotype, the frequency of drug resistance was calculated in each genotype (Table 3). CagA status was not significantly associated with metronidazole resistance (p-value = 1). Analysis of all strains revealed that genotypic resistance to metronidazole was similar in CagA positive strains and in CagA negative strains [62.9% (n = 44/70) vs 62.6% (n = 72/115)] (Table 3). By the combination of VacA and CagA genotypes, we remarked that the moderate virulent strains s1/m2/i1/d1-cagA negative, were the most commonly resistant to metronidazole with a rate of 84%, compared to the less virulent strains s2/m2/i2/d2-CagA negative with a rate of 58%, and the high virulent strains s1/m1/i1/d1-CagA positive with a rate of 47.1% (Table 3). We can deduce that metronidazole resistance was observed more in CagA-negative strains. Concerning the VacA gene, in strains with the VacA m2 allele, metronidazole resistance was more frequent.

**Table 3:** prevalence of metronidazole-resistant *H. pylori* strain according to CagA-VacA status

*H. pylori* strains	Metronidazole-resistance (%)	Metronidazole-susceptibility (%)	Total
CagA (+)	44 (62.9)	26 (37.1)	70
CagA (-)	72 (62.6)	43 (37.4)	115
s2/m2/i2/d2 CagA -	40 (58)	29(42)	69
s1/m2/i1/d1 CagA -	21(84)	4(16)	25
s1/m1/i1/d1 CagA +	16(47.1)	18(53)	34

## Discussion

**Epidemiology of metronidazole *H. pylori* resistance in Morocco**: in Morocco, the prevalence of *H. pylori* is very high so it is very important to know the rates of its resistance to antibiotics, especially when antibiotic resistance represents a serious public health problem in Morocco and around the world. The rate of primary resistance to metronidazole was documented as 40.1% in 2017 [2]. There is an increase in the rate of metronidazole resistance with (62.70%) found in our present study. Our result was similar to the rate found in China (63.8%) [21] and Algeria (67.5%) [22] but was much higher than that found in Europe (38.9%) [23] and Iceland (1%) [24], and lower than that found in Nigeria (99.1%) [25], Columbia (83%) [26] and in Iran (79%) [27].

Several authors have suggested that the prior use of metronidazole for other indications, such as gynecologic infections, could account for the resistance increase [5,28]. This could also be the case in Morocco. This would also explain the higher prevalence of metronidazole resistance in women that has been observed in several studies [21,22]. Our study, however, did not show a significant difference between men and women or a more apparent increase in women. The distribution of resistance according to age shows that the prevalence of metronidazole resistance is high in all age groups except in the age groups of 41-50 with no statistically significant difference between groups (p-value = 0.16) (Table 2). We have also noticed that the distribution of metronidazole resistance remains high among old people in our country (Table 2). Our results showed also that the living area is not a risk factor for *H. pylori* infection by resistant strains.

***H. pylori* resistance to Metronidazole and virulence factors**: *H. pylori* VacA and CagA virulence genes are the most important virulence-associated genes that play an important role in the pathogenesis of *H. pylori*-related gastrointestinal disease. It is evident now that the presence of *H. pylori* virulence genes and their different genotypic combinations in the strain colonizing a patient affects the development of gastric disease. Some characteristics of *H. pylori* strains have been associated with the progression of infection to more severe diseases [13,22]. Some genotypes, such as alleles s1 and m1 of the VacA gene and the presence of the CagA gene, are considered pathogenicity markers since they are associated with cytotoxin production and the induction of more intense epithelial lesions and inflammatory reactions [22]. The relation between virulence factors and antibiotic resistance is another important issue. In this study, we investigated the association of *H. pylori* virulence factors VacA and CagA with the resistance to metronidazole. In our study, no association was revealed between the cagA gene and the resistance to metronidazole. This result is inconsistent with other studies that suggest the statistical relation between drug resistance and CagA positive strains [28,29]. While by the combination of cagA with VacA genotypes, we remarked that the moderate virulent strains, s1/m2/ i1/d1-cagA negative, were the most resistant to metronidazole (84%), and the high virulent strains VacA s1/m1/i1/d1-cagA positive were the less resistant to metronidazole (47.1%). Whereas the less virulent strains VacA s2/m2/i2/d2 - CagA negative, were resistant to metronidazole with a rate of 58%.

We hypothesized that both the absence of CagA and the presence of the VacA m2 allele are two important reasons for the acquisition of more resistance to metronidazole. An Irish study revealed a high frequency of metronidazole resistance in CagA negative status. In contrast, they noted no association between VacA m allele and metronidazole resistance [30]. Another Dutch study was carried out which concluded that the non-virulent s2/m2 strains (which are mostly CagA negative) seem to be more resistant to therapy than the virulent ones s1/m1 and s1/m2 strains (which are mostly CagA positive) [31]. These results suggest that the CagA-positive strains seem to be more interested in growing faster and ignoring the mechanisms of antibiotics resistance. Contrary to Bachir et al, they found that virulence vacA or cagA genes have a positive impact on antibiotic resistance. They showed that infected patients with vacA s1m1/cagA positive virulent strains are at high risk to develop severe gastric lesions by *H. pylori* eradication failure [22]. While Dai *et al*. demonstrated that there was no difference in *H. pylori* metronidazole resistance between VacA m1 and VacA m2 strains [32]. Therefore, more studies are needed to understand the association between *H. pylori* virulence factors and antibiotics resistance. Our study has some limitations: Although metronidazole resistances were found in 62.7% of *H. pylori*-positive cases, these percentages could be still underestimated due to the presence of other mutations which were not studied in our study. On the other hand, to study the association between *H. pylori* resistance and virulence factors requires a larger population size.

## Conclusion

In conclusion, the prevalence of *H. pylori* metronidazole resistance is high in Morocco. This leads us to recommend either abandonment of standard metronidazole-based therapy as a first-line. Also, this study tested the hypothesis that the virulence factors are related to metronidazole resistance. Our results indicated that the relationship between metronidazole resistance and the availability of the cagA gene was not statistically significant. But interestingly, the strains exhibiting VacA m2 and the strains with a deleted CagA were more resistant to metronidazole in the study´s strains. Periodic monitoring of antimicrobial susceptibility is necessary to define the evolution of the resistance patterns of *H. pylori* in Morocco, and a study on a larger scale should be carried out to determine the metronidazole resistance profile of *H. pylori* strains isolated from patients throughout the whole country.

### What is known about this topic

- H. pylori infection is one of the most common chronic bacterial infections in the world;
- Metronidazole is often used in the eradication of H. pylori.

### What this study adds

- High prevalence of the resistance of H. pylori to metronidazole in a Moroccan population;
- Recommendation to stop the use of metronidazole-based treatment as first-line therapy for eradication of H. pylori in Morocco;
- The CagA and VacA virulent factors may influence H. pylori metronidazole resistance.
